# Supplementary material for: Patterns of Intron Gain and Loss in Fungi
Source: PLoS Biol. 2004 Nov 30;2(12):e422. doi: 10.1371/journal.pbio.0020422 (PMC532390; doi:10.1371/journal.pbio.0020422)
Supplement: Table S1 — Also available at http://genes.mit.edu/NielsenEtAl/. (4.3 MB ZIP). [file pbio.0020422.st001.zip › NielsenEtAl/html/426.html]

AN5196.1.NCU06921.1.MG08133.1.FG01399.1


```
 CLUSTAL W (1.82) Multiple Sequence Alignments - Introns Inserted


Sequence 1: NCU06921.1	630 aa
Sequence 2: FG01399.1	529 aa
Sequence 3: MG08133.1	542 aa
Sequence 4: AN5196.1	550 aa
Alignment Length: 658 aa
Number Identitical Residues: 208 aa
Alignment Score (without introns) 11781


MG08133.1 	------------------------------------------------------------
NCU06921.1	MEPNIRVIEHSLYCIVERQARNNRAWLNADHCQPLPHKQAESQLQSLVLAQPECDSNPVS
FG01399.1 	------------------------------------------------------------
AN5196.1  	---------------------------------------------MAALLGAAYDSSDDD
          	                                                   .  .:.  .

MG08133.1 	~---------------------------MSGFGEYAPNMVPTDALVVRDQAEPAHELVQY
NCU06921.1	1SSSVRLASIPSPQFHQAAAELNIIQIAMADFNEYPANLSVKDALIVRQVAEPDHAVVPY
FG01399.1 	~---------------------------MADFGEYPPNMAPQDALIVREQAEPDHAVVPF
AN5196.1  	~TPSVPAASAPKVVAAPEVNTEVWSLQSCQHKSTLSLTDLQDQSHMRMMLANTSSKALTY
          	 :.:   :: ..               :    .  . .    :: :    *:.    : :

MG08133.1 	TG--EDLARPKFGPSNPFRDDSSAAS--ASQKRKNVLTGFAEETFVSEHTFRSKHRAVER
NCU06921.1	TG--EDLSRPRLGPANPFRDDTDSTSTAVGTKRKNVLTGTAAETYISEHTFRSKHRAIER
FG01399.1 	TA--EDLSRPKAGPLNPFKDESN------VLKRKSVPTGHAEETFLSEHTFRSKHRAIER
AN5196.1  	NATYDDLSRPSQGPSNPFKSAGAGN----GLKRKNVPTGYAEAAAISESTFTAQHRTYQS
          	..: :**:**  ** ***:.   .       ***.* ** *  : :** ** ::**: : 

MG08133.1 	RGG--------------------PEREFQSGAEVKAEAARIRKTREGKGSALVAEGDGEY
NCU06921.1	GGG--------------------PAREQLGSTAKKAAAAKLRQQREGKGSATIADGDGAY
FG01399.1 	LGGR-------------------PERQYQTNAELKAEAARIRAGREDKGSAIIAEGPGSY
AN5196.1  	LGYTRNPTAPEQFVGNLDHAAQFGGRDVVQMKPSKEASAALRAKRQKKGDSSIVEGPGAY
          	 *   ..::...  .. . ::.   *:       *  :* :*  *: **.: :.:* * *

MG08133.1 	KGPWASYKSRQARGIEVELEGGGEELASDEEYEIVEVED-DEDEDVVESGAVVQAPAEAL
NCU06921.1	VGPWARYKKQ-----EYEEVNE-EELGSGEEYEIVEEEEGEEEEDVVESGTVVKAPTKNL
FG01399.1 	VGPWARYKKA-----EYEIVAEDEELASDEEYEIVEEEE-----EVVESGTIVKAPTKAL
AN5196.1  	LGPWAKYQDD-------DQVYD-EELGSDEELVEVDEDE----EEQEQVGSAPMPAMSKE
          	 **** *:.        :     ***.*.**   *: ::    .:  : *:   .. .  

MG08133.1 	ARRQQVEDLGDETTTFHGSELHDYQGRTYMH0VPQDLDIDLRKEPGSTTNYIPKKMVHQW
NCU06921.1	ARRKEVEEQGAETTTFHGTEEFDYLGRTYMH~VPQDLDISLTKDVGSVTNFIPKKHMHTW
FG01399.1 	EQRKEAENMGDERTEFHGSAETDYQGRTYMH~VPQDLGIDLRKEVGSVTNYIPRKQIHSW
AN5196.1  	ATDYQDDTSKVETTEFHGSEQYDYLGRTYMH~VPQDLDIDLKKPVGSIKNYVPKKLVHTW
          	    : :    * * ***:   ** ****** *****.*.* *  ** .*::*:* :* *

MG08133.1 	KHHT-KAITSLNFFPNSGHLLLSGSADSTVKIWDVYHQRELLRTYSGHSKSLSDTTFNTH
NCU06921.1	RHHAGKPITALQLFPKSSHLGLSGSTDSTIKIFDVYRNRELLRTYAGHSKAITDLSFNRD
FG01399.1 	KNHN-KAVTALQFFPTSGHLLLSASADTTVKIFDMYHDRELLRTYHGHSKALSDICFNTS
AN5196.1  	KSHT-KAITSLRFFPQAGHLLLSSAADGKAKIWDVYHSRELLRTFSGHSKAITDTDFHIT
          	: *  *.:*:*.:** :.** **.::* . **:*:*:.******: ****:::*  *:  

MG08133.1 	GDKFLSASFDRMMKLWDTETGKCIAKFTTGKTPHVVRFNPTSELGHEFVAGMSDNKIVQF
NCU06921.1	GTKFLSGSFDRWIKLWDTETGQCVNRFNTGKTPHVIKFNPSVDQGHEFLAGLSDNRIVQY
FG01399.1 	GTQFLSSSYDRMIKLWDTETGVCVNKFTTGKTPHVIKFNPGPEHANEFLAGMSDKKIVQF
AN5196.1  	GKTFLTASYDRQMKLWDTETGQCISRFSTGKTPHVIRFNPNPENSHEFLAGMSDKKIVQF
          	*  **:.*:** :******** *: :*.*******::***  : .:**:**:**::***:

MG08133.1 	DTRAGNETVQEYDHHLGPVNTITFCDDGRRFMTTSDDRTLRAWEYGFGVPIKYIA~EPYL
NCU06921.1	DSRAGADPVQEYDHHLGAINTLEFVDENRRFMSTSDDRSLRVWEYGIPVEIKTIS2EPDM
FG01399.1 	DIRT-KEVVQEYDHHLAAINTITFVDDNRRFMTTSDDKSLRAWDYGIPVPIKYIA~EPDM
AN5196.1  	DTRT-GEQVQEYDHHLAAINTLTFVDQNRRFISTSDDKSLRAWEYGIPVPIKFIA~EPYM
          	* *:  : ********..:**: * *:.***::****::**.*:**: * ** *: ** :

MG08133.1 	FAMTRAATHPSGKYVAYQSSDNQIVVYS-SNDKFRQNRKKSYRGHNNAGSAIDIDISADG
NCU06921.1	FALTKSTQHPSGKYVLYQCSDNSIVAYSSASDKFRQNRKKSWRGHNTAGSGIGLVCSPDG
FG01399.1 	YPMTRAAPHPSGKYVAYQSSDNQILVYG-ANDKFRQNRKKSYRGHNNAGLGIDLDCSPDG
AN5196.1  	FALTRAAAHPNGKYVAFQSGDNQIVVYG-ATDKFRQNRKKSFRGHNNAGYAIDLTISPDG
          	:.:*::: **.**** :*..**.*:.*. :.**********:****.** .*.:  *.**

MG08133.1 	QFLASGDTGG~YVAFWDWKSCKMFHKIQVED--GQVSCVKWHPQESSKVATGGASGIIKF
NCU06921.1	QFVASGDTGG~YVCFWDWKTCKMYHKIHADTSGGAINCVAWSEQETSKVFTAGAKGEIRM
FG01399.1 	QFLASGDSGG~YVCFWDWKTCKMYHKLKAGN--QAITSVKWHPQETSKVVTAGMDGEIRY
AN5196.1  	QFIASGDSAG2LFSWYLY------------------------------------------
          	**:****:.*  ..:: :                                          

MG08133.1 	WD
NCU06921.1	WD
FG01399.1 	WD
AN5196.1  	--
          	
```
